# Vortex‐Oriented Ferroelectric Domains in SnTe/PbTe Monolayer Lateral Heterostructures

**DOI:** 10.1002/adma.202102267

**Published:** 2021-07-03

**Authors:** Kai Chang, John W. D. Villanova, Jing‐Rong Ji, Souvik Das, Felix Küster, Salvador Barraza‐Lopez, Paolo Sessi, Stuart S. P. Parkin

**Affiliations:** ^1^ Max Planck Institute of Microstructure Physics Weinberg 2 06120 Halle Germany; ^2^ Beijing Academy of Quantum Information Sciences Beijing 100193 China; ^3^ Department of Physics University of Arkansas Fayetteville AR 72701 USA

**Keywords:** 2D ferroelectrics, group‐IV monochalcogenides, lateral heterostructures, molecular beam epitaxy, SnTe/PbTe monolayers

## Abstract

Heterostructures formed from interfaces between materials with complementary properties often display unconventional physics. Of especial interest are heterostructures formed with ferroelectric materials. These are mostly formed by combining thin layers in vertical stacks. Here the first in situ molecular beam epitaxial growth and scanning tunneling microscopy characterization of atomically sharp lateral heterostructures between a ferroelectric SnTe monolayer and a paraelectric PbTe monolayer are reported. The bias voltage dependence of the apparent heights of SnTe and PbTe monolayers, which are closely related to the type‐II band alignment of the heterostructure, is investigated. Remarkably, it is discovered that the ferroelectric domains in the SnTe surrounding a PbTe core form either clockwise or counterclockwise vortex‐oriented quadrant configurations. In addition, when there is a finite angle between the polarization and the interface, the perpendicular component of the polarization always points from SnTe to PbTe. Supported by first‐principles calculation, the mechanism of vortex formation and preferred polarization direction is identified in the interaction between the polarization, the space charge, and the strain effect at the horizontal heterointerface. The studies bring the application of 2D group‐IV monochalcogenides on in‐plane ferroelectric heterostructures a step closer.

## Graphical abstract

SnTe/PbTe monolayer lateral heterostructures are experimentally created through a two‐step molecular beam epitaxial growth process. A vortex‐oriented quadrant ferroelectric domain configuration is observed in the van der Waals 2D material heterostructure in this work.

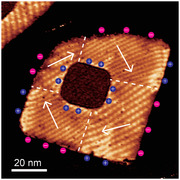

## 1 Introduction

After more than a decade of research, 2D materials continue to exhibit superior mechanical, electronic, spintronic, valleytronic, optical, thermal, magnetic, and ferroelectric performances in novel heterostructures and devices. Their weak interlayer coupling allows the relatively straightforward fabrication of vertical heterostructures by mechanical stacking of 2D materials. On the other hand, the creation of lateral heterostructures (LHSs), which are the elementary structures of the conducting channels in modern metal–oxide–semiconductor field‐effect transistor based microelectronics, is much less explored as it requires more complex growth and doping techniques. Encouraged by the potential outstanding performance and versatile tuning freedom that can emerge out of 2D LHSs, multiple experimental and theoretical studies have been carried out in this field.^[^ ^1^ ^]^ The earliest experimentally realized 2D LHSs were those between graphene and hexagonal boron nitride (hBN)^[^ ^2^, ^3^, ^4^, ^5^, ^6^ ^]^ grown by chemical vapor deposition (CVD), from which prototype field effect transistors (FETs) were demonstrated^[^ ^2^, ^3^, ^4^, ^5^ ^]^ Shortly later, a series of transition metal dichalcogenide (TMDC) monolayer (ML) LHSs, including combinations of MoS_2_, MoSe_2_, WS_2_, and WSe_2_, were prepared by one‐step or two‐step CVD methods.^[^ ^7^, ^8^, ^9^, ^10^, ^11^, ^12^ ^]^ All these TMDC LHSs display diode‐like electric current rectification effects. Meanwhile, prototype devices including photodiodes and complementary metal–oxide–semiconductor transistor inverters with high performance were fabricated^,[^ ^7^, ^10^, ^11^, ^12^ ^]^ Through either well‐controlled gas flow switching techniques or lithography assisted anion substitution, TMDC LHS superlattices with atomically sharp interfaces were created^[^ ^13^, ^14^, ^15^ ^]^ In addition, TMDC LHSs composed of only one material, but with varying thicknesses,^[^ ^16^, ^17^ ^]^ or dielectric environments^[^ ^18^ ^]^ across their interface, generated modifications of the electronic bandgap, rectification, and photovoltaic effects. Additional forms of 2D LHSs that combine materials with different spatial symmetries, such as graphene‐TMDC LHSs^[^ ^19^, ^20^, ^21^, ^22^ ^]^ hBN‐TMDC LHSs,^[^ ^19^ ^]^ graphene nanoribbon LHSs with varying doping^[^ ^23^ ^]^ or widths,^[^ ^24^ ^]^ metallic‐semiconducting TMDC LHSs,^[^ ^25^ ^]^ and group‐IV monochalcogenide‐dichalcogenide LHSs,^[^ ^26^ ^]^ have been created through various enhanced CVD approaches, such as mechanical‐exfoliation‐assisted CVD,^[^ ^19^ ^]^ seed‐promoted CVD,^[^ ^20^ ^]^ template‐growth defined by plasma etching,^[^ ^21^ ^]^ and thermally converting the chemical compositions.^[^ ^26^ ^]^

Recently, a series of 2D ferroelectric materials have been discovered,^[^ ^27^, ^28^ ^]^ allowing for new possibilities of next‐generation nonvolatile devices. With switchable spontaneous polarization, LHSs based on 2D ferroelectrics are naturally suitable for memories and low‐energy logic devices that can be easily tuned by external electric fields, such as edge‐contacted^[^ ^29^ ^]^ or lateral^[^ ^30^ ^]^ ferroelectric tunneling junctions and synaptic devices.^[^ ^31^ ^]^ Nevertheless, despite the promising potential of applications, experimental studies of LHSs containing 2D ferroelectric materials—and the concomitant understanding of their interfacial tuning effects—are still rare. Here, we report the molecular beam epitaxial (MBE) growth and scanning tunneling microscopy (STM) characterization of the LHS between two distinct group‐IV monochalcogenide MLs—an in‐plane polarized ferroelectric SnTe ML and a paraelectric PbTe ML. Besides demonstrating their atomically sharp interface and a type‐II band alignment by STM, we have discovered vortex‐oriented ferroelectric domain quadrants in SnTe with a preferred polarization direction at the SnTe/PbTe interface, which is ascribed to the charge transfer induced by the difference of work functions across the interface, according to first‐principles calculations and to Gundlach oscillations measured by STM.

## 2 Results

Group‐IV monochalcogenides are a category of materials with the chemical formula MX (M = Ge, Sn, Pb; X = S, Se, Te). Depending on the pressure, temperature, and chemical composition, the crystal structures of these materials can be orthorhombic, rhombohedral, or cubic. Only the orthorhombic phase has a layered bulk atomistic structure.^[^ ^32^ ^]^ Among the orthorhombic MX materials, SnTe,^[^ ^33^, ^34^ ^]^ SnSe,^[^ ^35^ ^]^ and SnS,^[^ ^36^, ^37^ ^]^ MLs have been experimentally demonstrated to be 2D ferroelectrics with an in‐plane polarization (space group *Pnm*2_1_) and ferroelectric behavior has been theoretically predicted for GeS and GeSe MLs^[^ ^38^, ^39^, ^40^, ^41^, ^42^ ^]^ On the other hand, PbTe MLs have been shown to be paraelectric semiconductors.^[^ ^33^ ^]^

**Figure 1:**
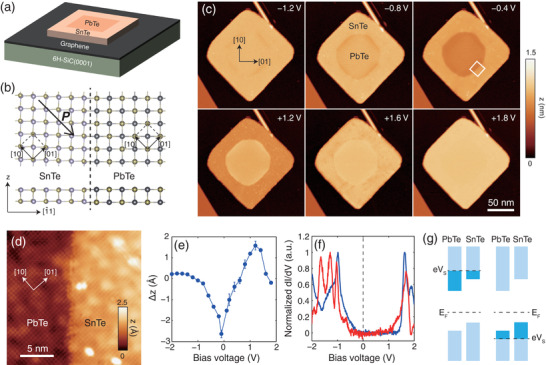
Type‐II band alignment in SnTe/PbTe ML LHSs. a) Schematic of the structure of the SnTe/PbTe LHS plates grown on graphitized 6H‐SiC(0001). b) Lattice structure of the LHS viewed from the *z*‐axis (top) and from the [11] direction (bottom), respectively. c) STM topography images of an LHS plate, with a sequentially increasing bias voltage *V*_s_ indicated at the upper‐right corner of each panel. The setpoint of the tunneling current *I*_t_ is 30 pA. d) Atom‐resolved topography image acquired at a section of interface along the [10] direction of SnTe, as indicated by the square in (c). Setpoint: *V*_s_ = −0.7 V, *I*_t_ = 100 pA. e) *V*_s_ dependence of the apparent height difference between SnTe and PbTe. The data points are obtained by averaging the apparent height of the flat terraces on SnTe and PbTe, respectively. f) d*I*/d*V* spectra acquired in the center of SnTe (blue) and PbTe (red) terraces. Setpoints: *V*_s_ = +3.0 V, *I*_t_ = 100 pA for *V*_s_ > 0 and *V*_s_ = −2.0 V, *I*_t_ = 100 pA for *V*_s_ < 0. g) The apparent height difference is induced by a type‐II band alignment: the darker color regions indicate the local density of states that contribute to the tunneling current. The left and right panels illustrate the situation with positive and negative *V*_s_, respectively. All the data were collected at 1.9 K except for that in (e), which were collected at 300 K.

Because of the similar lattice parameters and compatible crystalline structures of MX MLs, it is straightforward to conceive functional LHSs between these materials and multiple theoretical studies have proposed devices such as diodes and tunneling FETs.^[^ ^43^, ^44^, ^45^, ^46^, ^47^, ^48^, ^49^ ^]^ Nevertheless, because of the relatively strong interlayer coupling in orthorhombic MX materials, it is difficult to obtain large‐area MX ML flakes through mechanical exfoliation, making controlled growth or etching preparation methods a necessity.^[^ ^50^, ^51^ ^]^ Here we applied a two‐step MBE growth procedure to prepare SnTe/PbTe ML LHS nanoplates with PbTe in the core and SnTe at the perimeter, as schematically shown in **Figure** **1**a. In order to minimize the effect of substrate‐induced strain, these SnTe/PbTe LHS nanoplates were grown on Si‐terminated 6H‐SiC(0001) covered by epitaxial graphene, which has an extremely low surface energy and exerts almost no strain on the SnTe and PbTe lattices.^[^ ^33^ ^]^ In SnTe, the directions parallel or antiparallel to its in‐plane polarization are defined as <10> (armchair), while the in‐plane direction perpendicular to its polarization is defined as <01> (zigzag). In contrast to previous theoretical studies, which focus on the interfaces along <10> directions^[^ ^43^, ^44^, ^45^, ^46^, ^47^, ^48^, ^49^ ^]^ the SnTe/PbTe interfaces in our experiments mainly occur along the <11> directions (Figure 1b,c), which are also the preferred directions for the exposed edges in stand‐alone SnTe and PbTe ML nanoplates.^[^ ^33^ ^]^ Atom resolved STM topography imaging confirmed atomically sharp interfaces (Figure 1d). According to previous studies,^[^ ^32^ ^]^ the bright spots in the image are the Sn or Pb atoms at the topmost atomic layer.

Figure 1c showcases STM topography images acquired under increasing bias voltage *V*_s_ applied to the sample. Interestingly, our first‐principles calculations predict that the thickness of the SnTe ML is similar to that of PbTe ML, but the apparent heights of the SnTe ML (*z*_S_) and the PbTe ML (*z*_P_) show a dramatic and nonmonotonic *V*_s_ dependence, as summarized in Figure 1e. When *V*_s_ < −1.2 V, *z*_S_ and *z*_P_ are almost the same; as *V*_s_ increases, both *z*_S_ and *z*_P_ decrease, while *z*_P_ drops faster than *z*_S_, resulting in a negative Δ*z = z*_P_ − *z*_S_, which reaches a maximum value of −2.6 Å at *V*_s_ = −0.1 V. As *V*_s_ increases further, Δ*z* begins to increase and finally reaches a positive maximum value of 1.6 Å at *V*_s_ = +1.2 V and then decreases again until close to zero around *V*_s_ = +1.8 V. Such a nonmonotonic *V*_s_ dependence of Δ*z* implies a type‐II band alignment between the SnTe and PbTe MLs. Indeed, as displayed by the d*I*/d*V* spectra in Figure 1f, the conduction band minimum (CBM) of SnTe and PbTe MLs lie at +1.46 and +1.00 eV, respectively, while the valence band maximum (VBM) is at −0.21 and −0.53 eV, respectively, resulting in bandgaps of 1.67 eV for SnTe and 1.53 eV for PbTe, and a type‐II band alignment, as schematically illustrated in Figure 1g. The Fermi level of SnTe MLs agrees well with the previous study,^[^ ^33^ ^]^ implying a hole density of ≈10^–10^ cm^–2^. PbTe MLs are less p‐doped than SnTe MLs as their VBM lies at a lower energy. When the STM operates in constant current mode, the apparent height *z* is influenced by the integral of the sample's local density of states (LDOS) between 0 and *eV*_S_ (see the Supporting Information for a detailed discussion of the image formation mechanism). As Figure 1g illustrates, when *V*_S_ is higher than the PbTe's CBM, PbTe has more LDOS entering the region of integration, hence *z*_P_ > *z*_S_. In contrast, when *V*_S_ is lower than the SnTe's VBM, the SnTe's LDOS overwhelms that of PbTe in the region of the integral and *z*_S_ > *z*_P_ is observed. The measurement of apparent height is an effective approach to determine the type of band misalignment at room or higher temperatures, at which d*I*/d*V* spectra are difficult to be acquired because of large thermal drifts. Such a ferroelectric/paraelectric interface could generate interesting hysteretic current rectification effects in electric transport measurements, which are important for fabricating nonvolatile devices, for example, memristors.

**Figure 2:**
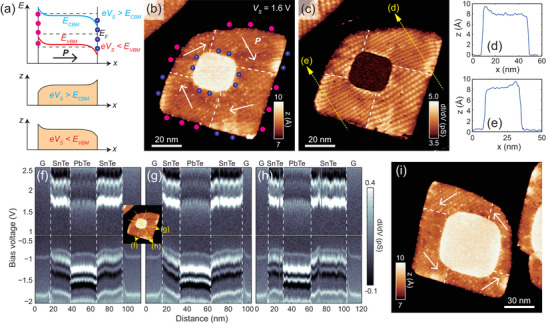
Vortex‐oriented domain quadrant configuration in SnTe/PbTe ML LHSs. a) Schematics of the polarization‐induced band bending on a SnTe ML and the corresponding apparent height profile with *eV*_s_ close to the CBM and VBM, respectively. b) STM topographic image of a LHS plate with a clockwise domain vortex. Setpoint: *V*_s_ = 1.6 V, *I*_t_ = 30 pA. c) A d*I*/d*V* image acquired simultaneously with (b); distinct graphite/SnTe ML moiré patterns are clearly resolved in all domains. The dashed lines in (b) and (c) indicate ferroelectric domain walls, which were identified from the sudden change of moiré pattern periods. d,e) Apparent height profiles measured along the dotted arrows in (c), confirming the in‐plane polarization indicated in subplot (b). f–h) Spatially resolved d*I*/d*V* spectra, acquired along the dotted arrows in the inset. Setpoints: *V*_s_ = +3.0 V, *I*_t_ = 100 pA for *V*_s_ > 0 and *V*_s_ = −2.0 V, *I*_t_ = 100 pA for *V*_s_ < 0. i) STM topography image of a LHS plate with a counterclockwise domain vortex configuration. Setpoint: *V*_s_ = 1.6 V, *I*_t_ = 30 pA. All the data in this figure were acquired at 1.9 K.

The electronic structure at the interface of the ferroelectric‐paraelectric LHS is not only determined by the intrinsic band alignment; it is also influenced by the distribution of ferroelectric domains in SnTe. First‐principles calculation suggests an in‐plane polarization of 2.3 × 10^–10^ C m^–1^ in a free‐standing SnTe ML,^[^ ^32^ ^]^ which corresponds to a bound charge density of 1.6 × 10^–10^ C m^–1^ at the free <11> edges of the plates. As **Figure** **2**a illustrates, the bound charges generate a depolarization field near the edges and interfaces, which manifests itself as an additional electronic band‐bending that can be directly imaged by the spatially resolved d*I*/d*V* spectra^,[^ ^32^, ^33^, ^35^ ^]^ Since positive (negative) bound charges create downward (upward) band‐bending, the direction of in‐plane polarization is determined from the change of apparent height when *V*_S_ is set close to the CBM or VBM. In Figure 2b, STM topography and d*I*/d*V* images of an LHS plate are displayed within a restricted height range to clearly reveal the domain structure within SnTe. The moiré pattern generated by the overlapping of SnTe and graphene lattices is resolved in the simultaneously acquired d*I*/d*V* image (Figure 2c). A configuration of domain quadrants can be inferred by observing the abrupt change of moiré pattern periods, implying a spatially rotating polarization direction for SnTe around the PbTe core. (A detailed analysis of the moiré pattern between the SnTe MLs and graphene can be found in the Supporting Information of Ref. ^[^ ^33^ ^]^.) The analysis of the apparent heights at the edges confirms that this is the case: the in‐plane polarization in the domain quadrants leads to a vortex‐oriented polarization of the SnTe ML perimeter in these LHSs. More specifically, all domain walls are charge‐neutral 90° “head‐to‐tail” walls, which is the dominating type in SnTe MLs^,[^ ^33^, ^34^ ^]^ The characterization of vortex‐oriented domain configurations is further supported by the spatially resolved d*I*/d*V* spectra acquired along three different directions across the LHS plate in Figure 2b, as shown in Figure 2f–h. When the path of the STM tip is perpendicular to the edge/interface, a clear band‐bending that is consistent with Figure 2a,b can be seen (Figure 2f,g); on the other hand, when the path of the STM tip is along the diagonal of the plate, i.e., perpendicular to the polarization, no significant band‐bending is resolved (Figure 2h). It is worth noting that temperature‐driven polarization vortices in group‐IV monochalcogenide MLs have also been predicted by theory, but these polarization structures featuring a Berezinskii–Kosterlitz–Thouless phase transition have much smaller spatial‐ and time‐scales.^[^ ^52^, ^53^ ^]^

The vortex‐oriented quadrant domain configuration helps reduce the elastic energy in these 2D nanostructures. Despite the fact that vortex‐oriented ferroelectric domains^[^ ^54^, ^55^, ^56^, ^57^ ^]^ and nearly continuously rotating vortices^[^ ^58^, ^59^ ^]^ have been discovered in multiple ferroelectric nanostructures, this is the first report of static quadrant vortices in van der Waals 2D ferroelectrics, to the best of our knowledge. Additionally, similar amounts of clockwise (Figure 2b) and counterclockwise (Figure 2i) domain vortices have been observed in different plates on the same sample, implying the absence of any chirality preference in the domain formation process, as expected from the symmetry of the heterostructures. There might be possibility of experimentally engineering the chirality of vortices by, for example, growing the heterostructure on a chiral or magnetic substrate.^[^ ^60^ ^]^

**Figure 3:**
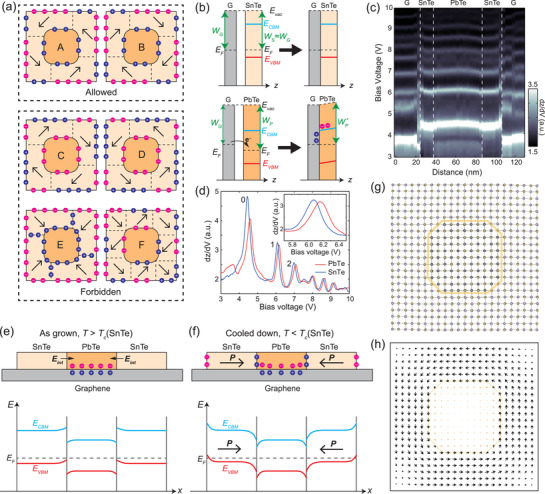
Preferred polarization directions in SnTe/PbTe ML LHSs and its interpretation. a) Schematic of several domain configurations, among which only the two types in panels A and B are allowed in the LHSs. b) Schematic of the charge transfer at the graphene‐SnTe and graphene‐PbTe interfaces. c) d*z*/d*V* spectra acquired across the diagonal of a square‐shaped LHS nanoplate displaying Gundlach oscillations (see the Supporting Information for more details). d) The d*z*/d*V* spectra of SnTe and PbTe acquired in the center of the terraces, in order to avoid the influence of band bending at the edges and interfaces. e,f) Interpretation of the preferred “inward‐pointing” polarization directions. The upper panels illustrate the cross‐section of a LHS plate, with SnTe in the paraelectric (e) and ferroelectric (f) phases, respectively. The lower panels are the corresponding band alignment diagrams. g) First‐principles calculated relaxed geometry of a SnTe/PbTe ML LHS nanoplate, approximately square and 7.3 nm in width. The starting geometry was oriented with the domain configuration that schematically shown in panel A of (a). h) In‐plane orientation of the dimer tilts (roughly proportional to local polarization) across the nanoplate. Each arrow indicates the averaged tilts of the Te‐Sn/Pb dimers within one unit cell.

An interesting phenomenon was observed while investigating multiple LHS plates: among many possible domain configurations, only those with an “inward‐pointing” (from SnTe to PbTe) polarization vortex‐oriented quadrant domain configuration (either clockwise or counterclockwise, as shown in panels A and B in **Figure** **3**a) are allowed, manifesting two important features: i) the polarization‐induced bound charges at the SnTe/PbTe ML interface are always positive, in contrast to the cases shown in panels C and D, and ii) at the corners of the interface (short <10> edges), the polarization of the SnTe ML is always parallel to the interface, in contrast to the case shown in panel E. Combining first‐principles calculations and STM experiments, we ascribe feature (i) to the difference in the work functions of SnTe and PbTe MLs and feature (ii) to the interfacial strain effect. We will elaborate these mechanisms below.

According to our first‐principles calculations (see the Experimental Section) with spin–orbit coupling taken into consideration, the work functions of bilayer graphene, SnTe and PbTe are *W*_G_ = 4.16 eV, *W*_S_ = 4.17 eV, and *W*_P_ = 4.25 eV, respectively. Therefore, there should be almost no charge transfer at the graphene‐SnTe interface, while electrons flow from graphene to PbTe and leave PbTe negatively charged at the graphene‐PbTe interface, as illustrated in Figure 3b. After charge rebalance, the work function at the SnTe and PbTe surfaces, ${W^{\prime }}_{S}$ and ${W^{\prime }}_{P}$, can be experimentally scrutinized by analyzing the sequence of the resonant states emerging within the tunneling junction when operated in the field emission mode, i.e., the so‐called Gundlach oscillations.^[^ ^61^ ^]^ These resonances are standing‐wave states caused by the constructive interference of electrons within the triangular potential well defined by the tunneling junction. They result in the emergence of well‐defined peaks, as shown in Figure 3c,d, in constant current spectroscopy curves (d*z*/d*V*) acquired in closed feedback loop conditions. The d*z*/d*V* spectra acquired at the surfaces of PbTe and SnTe have similar shapes but are shifted in energy. According to the energy shift of the first‐order peaks collected from five different LHS nanoplates, the work function difference, $\Delta W\;={W^{\prime }}_{P}\;-{W^{\prime }}_{S}$ = 90 ± 28 meV, agrees well with first‐principles calculations (the first‐order peaks were used because zeroth‐order peaks have been reported to not reflect well Δ*W* ^[^ ^62^ ^]^). Another analysis method also yields similar results (see the Experimental Section).

The value of Δ*W* well accounts for the preferred “inward‐pointing” polarization at the LHS interfaces. Right after the growth, when SnTe is paraelectric (*T* > *T*_C_), the negatively charged PbTe creates an interfacial electric field *E*_int_ that points from SnTe to PbTe and results in mild downward band‐bending in PbTe and upward band‐bending in SnTe, as shown in Figure 3e. Since *E*_F_ − *E*_VBM_ in PbTe is larger than that in SnTe, the downward band‐bending in PbTe is more pronounced and can be resolved from topography images acquired at room temperature (see the Supporting Information). While, as the sample is cooled down, the SnTe ML enters its ferroelectric phase and a spontaneous polarization emerges (Figure 3f). At the interface, *U*∝ −*P* · *E*_int_, in which *U* is the dipole energy in an electric field, so that a smaller angle θ between *P* and *E*_int_ is energetically more favorable. Therefore, at the <11> interfaces, *θ *= π/4 (“inward‐pointing”) is more stable than *θ *= 3π/4 (“outward‐pointing”). This is the first time that a preferred polarization direction has been observed at the interface of a 2D ferroelectric LHS. Such an effect might be universal in 2D ferroelectric LHSs and will be useful for constructing biased ferroelectric devices.

Having discussed the reason why the domain configurations in panels C and D in Figure 3a are not preferred, we now turn to the case shown in panel E, where the polarization in SnTe is also “inward‐pointing” but perpendicular to the <10> edges at the corners of the interface, i.e., the polarization is no longer vortex‐oriented. The SnTe ML has lattice parameters of *a*_1_ = 4.58 Å and *a*_2_ = 4.44 Å,^[^ ^33^ ^]^ while the lattice constant of the square lattice of PbTe is *a* = 4.60 ± 0.03 Å, according to our moiré pattern analysis (see the Supporting Information). When *a*_1_ of SnTe is parallel to the <10> edges of PbTe, there is a lattice mismatch of only −0.4%, while for the case *a*_1_ perpendicular to PbTe <10> edges, the lattice mismatch is as large as −3.5%. As the polarization is along the *a*_1_ direction, the case shown in panel E suffers from larger lattice elastic energy than those in panels A and B. Furthermore, the domain orientation in panel E also introduces positively charged “head‐to‐head” 90° domain walls, which also introduces extra depolarization field and raises the electrostatic energy. Therefore, the domain configuration in panel E is not favored, albeit the polarization is also “inward‐pointing.”

Density functional theory calculations using the SIESTA code have also confirmed the vortex‐oriented polarization preference. Two unsupported SnTe/PbTe ML LHS nanoplates containing 1058 atoms were initialized with counterclockwise “inward‐pointing” polarization and clockwise “outward‐pointing” polarization, respectively (Figure 3g). Relaxation of the atomic positions for all but the core 98 atoms led to ground state configurations that maintain the vortex‐oriented polarization. The case of counterclockwise vortex is shown in Figure 3h. (Further details are available in the Experimental Section.) Limited by the computing power, the width of the simulated plate (7.3 nm) is one order of magnitude smaller than those in the experiments. The simulated vortex‐orientation of polarization is in a more continuous fashion, instead of the quadrant domains in our experiments, most likely because of a quantum‐confinement effect, which is an interesting topic for further experimental and theoretical studies.

## 3 Discussion

Domain configurations with more than four domains are not preferred because there will inevitably be some interfaces with “outward‐pointing” polarization or negative bound charges. Panel F in Figure 3a shows an example with eight domains. In this case, although large strain is avoided, the negative bound charge on half of the interface increases the electrostatic energy and makes this configuration unstable. Configurations with one, two, or three domains also introduce either extra elastic or electrostatic energies compared with the vortex‐oriented quadrant domains, making the latter the most stable domain configuration in the SnTe/PbTe ML LHSs.

## 4 Conclusion

We have synthesized lateral heterojunctions from ferroelectric SnTe MLs and paraelectric PbTe MLs, with the PbTe ML at the center of these nanoplates grown through a two‐step MBE process. In order to minimize electrostatic and elastic energies, the SnTe ML at the perimeter of these nanoplates develop in‐plane vortex‐oriented quadrant domains whose polarizations point inwards, as demonstrated by STM experiments and consistent with the band alignment expected from calculated work functions. The novel vortex‐oriented lateral heterostructures hereby demonstrated highlight the possibilities of engineering the polarization state of 2D ferroelectrics with an in‐plane polarization and open unforeseen opportunities for use of these novel materials.

## 5 Experimental Section

### Sample Preparation and Characterization

The SnTe/PbTe LHSs were grown in a home‐built, ultrahigh vacuum MBE chamber with a background pressure of 5 × 10^−10^ mbar. To obtain graphitized surfaces, the Si‐face phosphor doped 6H‐SiC(0001) substrates were annealed following the recipe described in ref. ^[^ ^33^ ^]^. PbTe and SnTe molecular fluxes were used from Knudson cells (MBEKomponenten) hosting 99.999% PbTe or SnTe granules. The LHSs were grown through a two‐step deposition process. First, PbTe ML cores were deposited at a substrate temperature of 185 °C and a PbTe evaporator temperature of 420 °C; the deposition time was 9.5 min. Then with the same substrate temperature, SnTe was evaporated using an evaporator temperature of 400 °C for 8 min. The as‐grown samples were immediately transferred into a room temperature STM (Omicron Company, model VT‐STM‐XT) through a vacuum suitcase pumped by a lithium‐battery‐powered ion getter pump, whose base pressure was better than 1 × 10^−9^ mbar. The pressure in the STM chamber was 1 × 10^−10^ mbar. Pt/Ir tips calibrated on a Au(111) single crystal surface were used in the room temperature STM measurements. Then the samples were transferred to a low‐temperature STM (Scienta Omicron) for d*I*/d*V* and d*z*/d*V* spectra measurements, again with the vacuum suitcase. The d*I*/d*V* spectra were acquired at 1.8 K and the d*z*/d*V* spectra were acquired at 77 K.

For the measurements of Gundlach oscillations, the energy of the peaks *E*_n_ is given by^[^ ^62^, ^63^ ^]^

(1) $$E_{n}=\;W\;+\;{\left(\frac{\hbar^{2}}{2m}\right)}^{1/3}{\left(\frac{\;3\pi eF}{2}\right)}^{2/3}\;{\left(n\;-1/4\right)}^{2/3}$$

where *W* is the work function, *F* is the electric field in the tunneling junction, and *n* = 1, 2, 3, … the quantum number (note that *n* = 0 is not applied here). To obtain the work function difference, the sequence of the resonant states was mapped across the SnTe/PbTe heterostructures along three different directions (see the Supporting Information for the exact traces). Results were analyzed in two distinct ways, namely: i) extracting the work functions for both SnTe (${W^{\prime }}_{S}$) and PbTe (${W^{\prime }}_{P}$) by fitting the peaks according to Equation (1) and subsequently calculating their difference $\Delta W\;={W^{\prime }}_{P}\;-{W^{\prime }}_{S}$ and ii) measuring the relative energy shift for the first peak (*n* = 1): Δ*W* = *E*_1,P_ − *E*_1,S_.

Note that work functions can also be experimentally obtained through the derivative of the logarithmic tunneling current *I*_t_ with respect to the distance between the electrodes *z*, i.e., dln *I*_t_/d*z*, as shown by Binning and Rohrer in a seminal work.^[^ ^64^ ^]^ However, the analysis of field emission resonance states was demonstrated as being capable of determining work functions and their local variations with significantly higher precision,^[^ ^62^, ^63^ ^]^

Due to the high electric fields present in the tunneling junction in this spectroscopic mode, frequent tip changes were needed. To exclude the influence of tip‐induced artifacts, the Pt/Ir tip was spectroscopically characterized on a Ag(111) surface before as well as after the measurements. Moreover, five SnTe/PbTe heterostructures were analyzed where each heterostructure was measured using a different microtip (see the Supporting Information). Averaging the results from five different nanoplates, these two methods yielded Δ*W* = 118 ± 42 meV and 90 ± 28 meV, respectively. Both results consistently indicated a higher work function for PbTe, in line with the theoretical calculations.

### Computational Methods

Density functional theory calculations were performed using the Vienna Ab initio Simulation Package,^[^ ^65^, ^66^ ^]^ within the Perdew–Burke–Ernzerhof generalized‐gradient approximation^[^ ^67^ ^]^ for the exchange‐correlation functional with spin–orbit coupling. Projector augmented wave pseudopotentials^[^ ^68^ ^]^ were used with an energy cutoff of 500 eV and a 15 × 15 × 1 Monkhorst–Pack *k*‐point mesh. Additional calculations were performed with the HSE06 functional^[^ ^69^ ^]^ in order to reliably estimate the bandgaps and work functions. The SIESTA code^[^ ^70^ ^]^ was employed for the density functional theory calculations on the SnTe/PbTe nanoplate. The system of 1058 atoms was initialized with a counterclockwise “inward‐pointing” polarization for the SnTe perimeter with a 37 Å vacuum region in both in‐plane directions and a 20 Å out‐of‐plane lattice vector length to prevent interaction with periodic copies. While holding fixed the central 98 atoms in the PbTe core, the rest of the nanoplate was relaxed until forces were less than 0.05 eV Å^−1^.

The work function, *W*, was calculated from *W* = *E*_vac_ − *E*_F_, where *E*_vac_ is the vacuum level and *E*_F_ is the Fermi level.

## Conflict of Interest

The authors declare no conflict of interest.

## Supporting information

Supporting Information

## Data Availability

The data that support the findings of this study are available from the corresponding author upon reasonable request.
