# Supplementary material for: Vortex‐Oriented Ferroelectric Domains in SnTe/PbTe Monolayer Lateral Heterostructures
Source: Adv Mater. 2021 Jul 3;33(32):2102267. doi: 10.1002/adma.202102267 (PMC11469125; doi:10.1002/adma.202102267)
Supplement: Supplementary file 1 — Supporting Information [file ADMA-33-2102267-s001.pdf]

© 2021 Wiley-VCH GmbH

ADVANCED MATERIALS

Supporting Information

for *Adv. Mater.,* DOI: 10.1002/adma.202102267

Vortex-Oriented Ferroelectric Domains in SnTe/PbTe Monolayer Lateral Heterostructures

Kai Chang, John W. D. Villanova, Jing-Rong Ji, Souvik Das, Felix Küster, Salvador Barraza-Lopez,* Paolo Sessi,* and Stuart S. P. Parkin**

Copyright Wiley-VCH GmbH, 2021

Supporting Information

Vortex-Oriented Ferroelectric Domains in SnTe/PbTe Monolayer Lateral Heterostructures

Kai Chang, John W. D. Villanova, Jing-Rong Ji, Souvik Das, Felix Küster, Salvador Barraza-Lopez, Paolo Sessi, Stuart S. P. Parkin

This Supporting Information includes:
Supplementary texts
Figures S1-S4
Table S1

The mechanism of the apparent height in STM topography images

At a constant tunneling barrier width, the tunneling current between the STM tip and the sample surface can be written as

$$I_t = \frac{4\pi e}{\hbar}\int_{-\infty}^{+\infty}[f(-eV_S+E)-f(\epsilon)]\rho_S(-eV_S+E)\rho_T(E)|M|^2 dE, \quad (1)$$

in which $f(-eV_S+E)$ and $f(E)$ are the Fermi distribution of the sample and the STM tip, $\rho_S(-eV_S+E)$ and $\rho_T(E)$ are the density of states of the sample and the STM tip, and M is a tunnel matrix element. Here eV_S denotes the difference between the Fermi energies of the sample and the STM tip when a bias voltage V_S is applied to the sample. Since the energy scale in the characterizations above is much larger than the thermal fluctuation energy k_BT (0.36 meV at 4.2 K and 25.8 meV at 300 K), the Fermi distribution can be approximated as a step function, and $f(-eV_S+E)-f(E)=1$ only when $E \in [0, eV_S]$. If we assume that the density of states of a well-treated and calibrated STM tip is featureless, and ignore the tunnel matrix element effect, Eq. (1) can be simplified as

$$I_t \propto \int_0^{eV_S}\rho_S(-eV_S+E)\,dE. \quad (2)$$

When the STM operates in constant current mode, the tip is raised (dropped) when the detected I_t is higher (lower) than the setpoint, thus the apparent height z, which is the trace of the STM tip when scanning on the sample surface, is influenced by the integral of the sample's local density of states (LDOS) between 0 and eV_S. As Figure 1f illustrates, when V_S is higher than PbTe's CBM, PbTe has more LDOS entering the region of integration, hence $z_P > z_S$. In contrast, when V_S is lower than SnTe's VBM, SnTe's LDOS overwhelms that of PbTe in the region of integral and $z_S > z_P$ is observed.

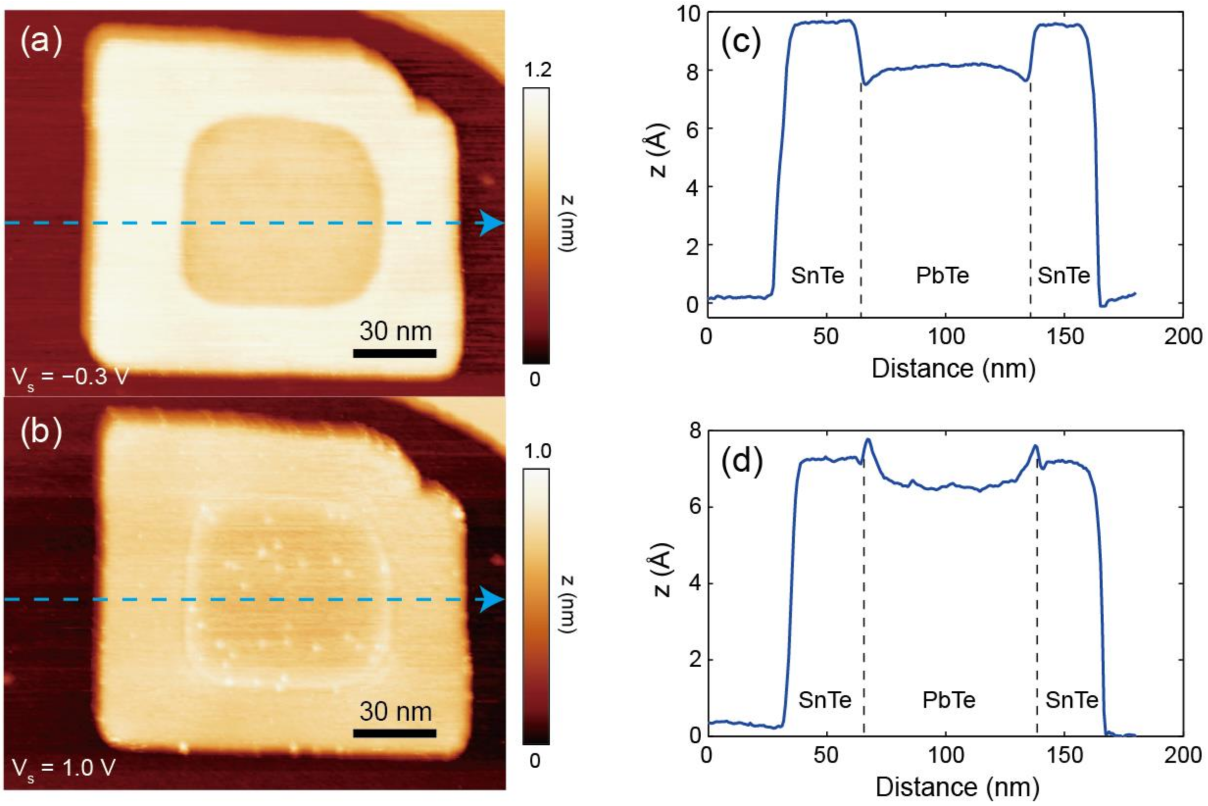

Figure S1. Band bending of PbTe at room temperature. (a),(b) STM topography images of a LHS nanoplate, acquired at room temperature. Setpoints, $V_s = -0.3$ V, $I_t = 2$ pA for (a); $V_s = 1.0$ V, $I_t = 2$ pA for (b). (c),(d) Apparent height profiles extracted along the dashed arrows in (a) and (b), respectively. It can be clearly seen that at the interface, the apparent height of PbTe reduces at a negative bias voltage close to PbTe's VBM, and increases at a positive bias voltage close to PbTe's CBM. This implies a downward band-bending induced by the electron doping, consistent with the interfacial charge transfer deduced from the work function calculation and measurements.

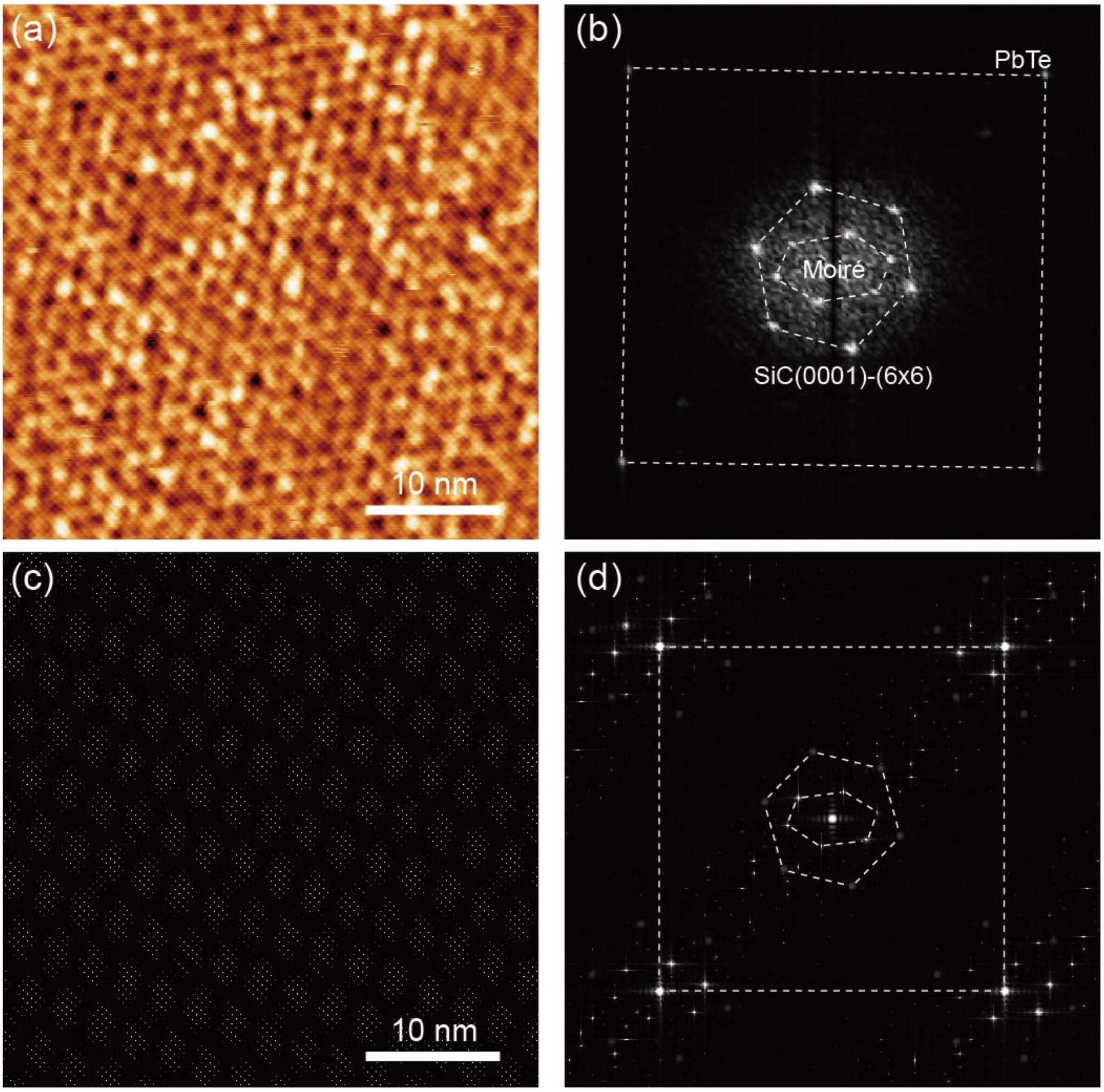

Figure S2. Determining the lattice constant of PbTe from a moiré pattern simulation. (a) An atom resolved STM image acquired at the surface of a PbTe ML plate, with no LHS grown. Setpoint: $V_s = -0.6$ V, $I_t = 50$ pA. (b) The Fourier transformed image of (a). Except from the Bragg spots of the square PbTe lattice, there are two superstructures: the regular hexagon is from the (6 × 6) reconstruction on a SiC(0001) surface, and the distorted hexagon is from the moiré pattern between the PbTe and graphene lattices. (c) The moiré pattern simulated from a MATLAB code superposing a square lattice (a = 4.60 Å for PbTe) and two hexagonal lattices (a = 2.46 Å for graphene, and a = 18.48 Å for the reconstruction of SiC). There is a rotation angle of 16 ° between the basis of the PbTe and graphene lattices. (d) Fourier transformed image of (c). The moiré pattern in (b) has been quantitatively well reproduced. The parameter space has been extensively searched and a = 4.60 Å for PbTe gives the best fitting. The uncertainty of ±0.03 Å comes from the deflection at which significant disagreement between the simulation and the experimental moiré pattern can be observed.

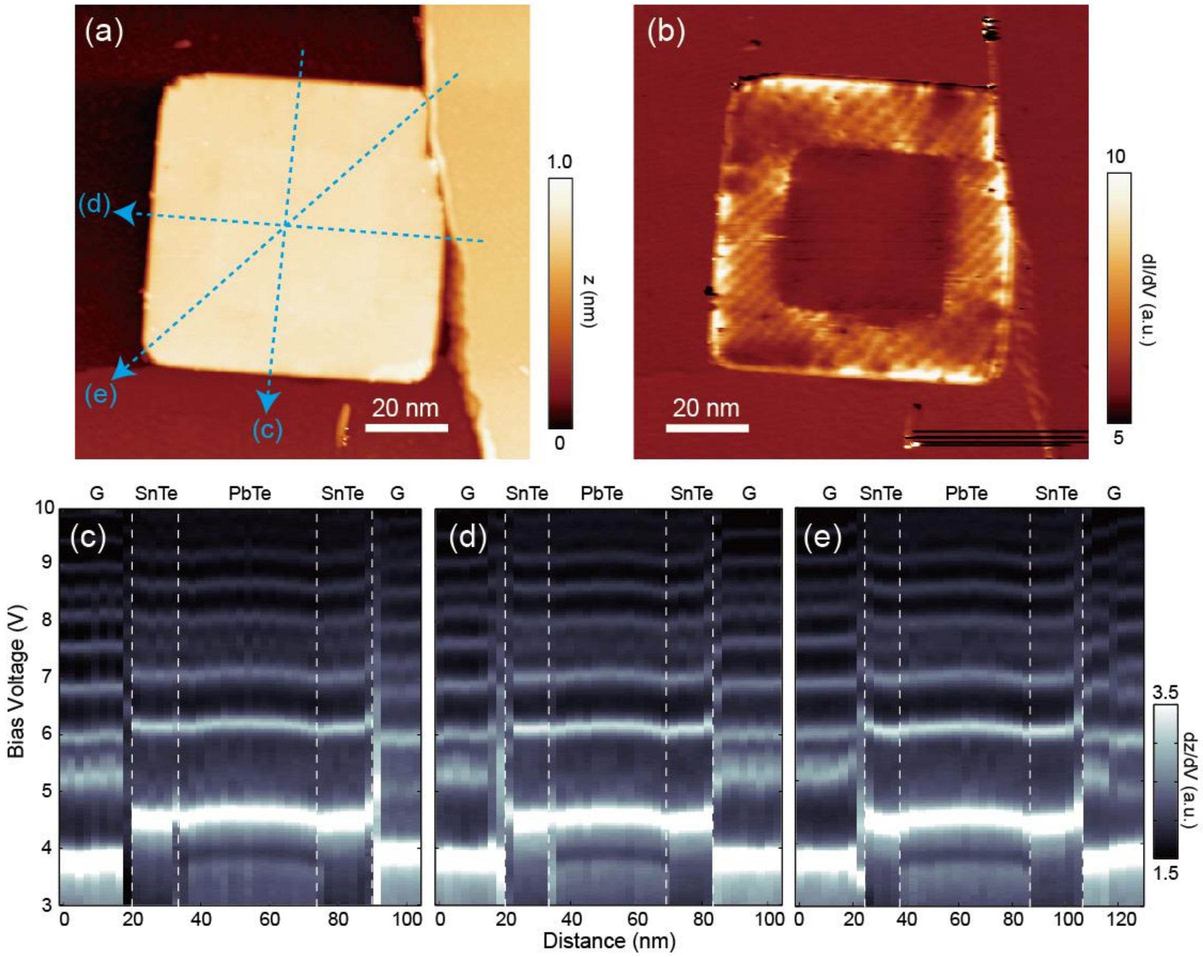

Figure S3. dz/dV spectra acquired along different directions across a square-shaped LHS plate. (a) STM topography image of the LHS plate. Setpoint: V_s = 1.7 V, I_t = 30 pA. (b) Simultaneously acquired dI/dV image (feedback loop closed). V_{mod} = 20 mV. (c)-(e) dz/dV spectra acquired along the dashed arrows indicated in (a).

Plate No.	From fitting (meV)	From n = 1 peak (meV)
**1**	130	110
**2**	50	50
**3**	160	110
**4**	110	70
**5**	140	110
**Average**	118	90
**Standard deviation**	42.07137	28.28427

Table S1. Statistics of the work function difference $\Delta W = W_P' - W_S'$ obtained from two different methods (see Experimental Section).

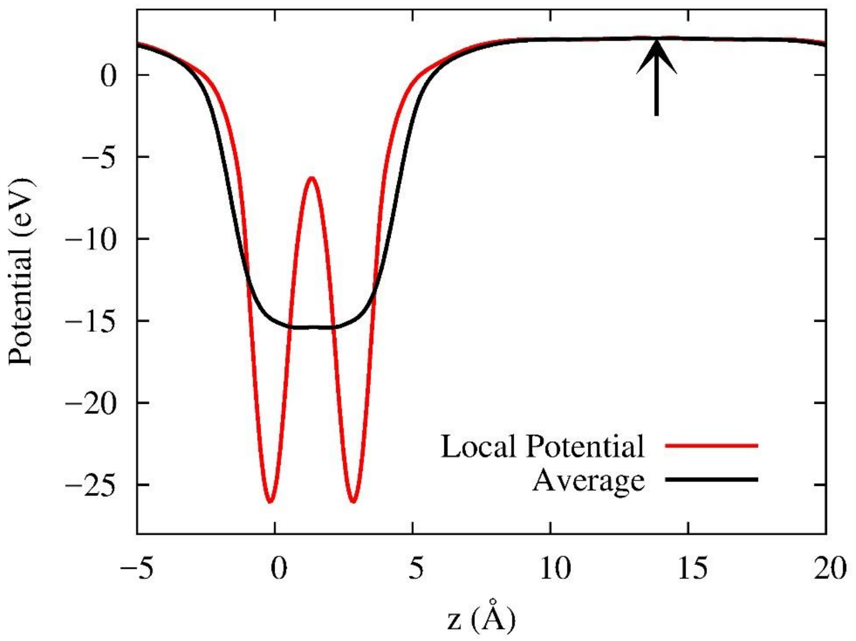

Figure S4. **Calculated local potential in a SnTe monolayer.** The local potential and its simple moving average are shown for a SnTe monolayer in a system with an out-of-plane lattice vector length of 25 Å. The arrow indicates the middle of the vacuum region where the average local potential provides the vacuum level in the calculation of the work function.

The vacuum level necessary to calculate the work function from VASP calculations is extracted from the LOCPOT file. Figure S4 demonstrates the macroscopically averaged local potential of a SnTe monolayer with nearly 22 Å of vacuum between periodic copies. Each value of the potential corresponds to a planar average over 72 $\times$ 72 points, and the simple moving average is performed with a window 3 Å in size.
